# Untargeted metabolomic analysis for the clinical screening of inborn errors of metabolism

**DOI:** 10.1007/s10545-015-9843-7

**Published:** 2015-04-15

**Authors:** Marcus J. Miller, Adam D. Kennedy, Andrea D. Eckhart, Lindsay C. Burrage, Jacob E. Wulff, Luke A.D. Miller, Michael V. Milburn, John A. Ryals, Arthur L. Beaudet, Qin Sun, V. Reid Sutton, Sarah H. Elsea

**Affiliations:** Department of Molecular and Human Genetics, Medical Genetics Laboratory, Baylor College of Medicine, One Baylor Plaza, NAB2015, Houston, TX 77030 USA; Metabolon Inc., Durham, NC USA

## Abstract

Global metabolic profiling currently achievable by untargeted mass spectrometry-based metabolomic platforms has great potential to advance our understanding of human disease states, including potential utility in the detection of novel and known inborn errors of metabolism (IEMs). There are few studies of the technical reproducibility, data analysis methods, and overall diagnostic capabilities when this technology is applied to clinical specimens for the diagnosis of IEMs. We explored the clinical utility of a metabolomic workflow capable of routinely generating semi-quantitative z-score values for ~900 unique compounds, including ~500 named human analytes, in a single analysis of human plasma. We tested the technical reproducibility of this platform and applied it to the retrospective diagnosis of 190 individual plasma samples, 120 of which were collected from patients with a confirmed IEM. Our results demonstrate high intra-assay precision and linear detection for the majority compounds tested. Individual metabolomic profiles provided excellent sensitivity and specificity for the detection of a wide range of metabolic disorders and identified novel biomarkers for some diseases. With this platform, it is possible to use one test to screen for dozens of IEMs that might otherwise require ordering multiple unique biochemical tests. However, this test may yield false negative results for certain disorders that would be detected by a more well-established quantitative test and in its current state should be considered a supplementary test. Our findings describe a novel approach to metabolomic analysis of clinical specimens and demonstrate the clinical utility of this technology for prospective screening of IEMs.

**Electronic supplementary material:**

The online version of this article (doi:10.1007/s10545-015-9843-7) contains supplementary material, which is available to authorized users.

## Introduction

Inborn errors of metabolism (IEMs) are inherited disorders typically caused by recessive mutations in genes encoding metabolic enzymes or transmembrane transporters. The list of recognized IEMs numbers in the hundreds and spans a wide clinical spectrum. Multiple specimen types and analytic approaches are currently required to screen for the compendium of IEMs (Burton 1998; Scriver 2001; Lanpher et al 2006). IEMs have been the focus of clinical investigation for over a century, with many now routinely screened for at birth (Seymour et al 1997; Schulze et al 2003; Scriver 2008). Yet, novel IEMs continue to be discovered, assisted in recent years by whole exome sequencing (Yu et al 2013; Thevenon et al 2014).

The study of metabolomics can involve the identification of small molecules in biological fluids with the aim of providing a complete view of metabolic status and uncovering metabolic pathway perturbations (Goodacre et al 2004; Werner et al 2008). Many prior metabolomic analyses of IEM patient specimens have focused on targeted mass spectrometry (MS) based approaches capable of providing absolute quantitation for a subset of predetermined analytes (Pitt et al 2002; Kuhara 2005; Janeckova et al 2012). Untargeted MS-based and therefore, non-quantitative metabolomics analysis has the potential advantage of detecting a much wider range of metabolites and disorders in a single test with a possible tradeoff of false negatives resulting from missed analyte identifications or in cases where small changes in concentration may be diagnostic. As MS analytics and associated technologies have matured, untargeted metabolomic studies have grown more precise and comprehensive, now allowing the identification of hundreds of unique plasma metabolites in a single analysis (Evans et al 2009; Psychogios et al 2011; Trushina et al 2013). Untargeted metabolomics studies of IEMs have been used to expand the range of disease-associated metabolites and to provide additional diagnostic information when paired with concurrent quantitative population screening (Wikoff et al 2007; Denes et al 2012).

As an initial test of the possibility that an untargeted metabolomics assay might allow sensitive and specific diagnosis of a wide range of IEMs, we analyzed 190 plasma specimens using an untargeted metabolomic workfow based on three separate mass spectrometry platforms run in parallel. Importantly, we approached this study as if it were a prospective screen for novel disorders; therefore we did not pool specimens or rely on cohort analyses to detect significant analyte changes. In this report we describe our results regarding the reproducibility of metabolomic findings and explore the diagnostic capabilities of this platform through the study of 21 different IEMs.

## Methods

### Sample collection

All procedures followed were in accordance with the ethical standards of the U.S. Department of Health and Human Services and were approved by the Baylor College of Medicine Institutional Review Board in accordance with the Helsinki Declaration of 1975, as revised in 2000. This study was approved with a waiver of informed consent.

Specimens used in metabolomic testing were collected from residual patient samples in our clinical biochemical genetics laboratory. All blood samples were initially preserved in lithium heparin at the site of collection, and plasma was isolated and shipped to our laboratory frozen. After clinical testing was complete, samples were stored in −20 °C for 1–9 months prior to metabolomic testing. For IEM patient samples, specimens were selected based on existing stocks in our laboratory with the goal of maximizing the diversity of diagnoses in our sample set. Clinical diagnoses were previously confirmed by biochemical and/or molecular genetic analyses in all cases; nearly all patients were undergoing clinical management for their disorder at the time of sampling. The undiagnosed patient specimens were also selected from our laboratory stocks but were prescreened to remove individuals with any biochemical findings from clinical testing that were interpreted as evidence of an IEM or of total parenteral-nutrition (TPN) at the time of sampling.

#### Metabolomic analysis

Metabolomic profiling was performed using three separate mass spectrometry platforms run in parallel essentially as described previously (Evans et al 2009). Starting with 100 μl of plasma, small molecules were extracted in an 80 % methanol solution containing four standards (tridecanoic acid, 4-Cl-phenylalanine, 2-flurophenylglycine, and d6-cholesterol) used to monitor extraction efficiency. Clarified supernatant was split into three aliquots and dried under N_2_. Additional internal standards (Standards for negative ion mode analyses included d7-glucose, d3-methionine, d3-leucine, d8-phenylalanine, d5-tryptophan, Cl-phenylalanine, Br-phenylalanine, d15-octanoic acid, d19-decanoic acid, d27-tetradecanoic acid, and d35-octadecanoic acid. Standards for positive ion mode analyses included d7-glucose, fluorophenylglycine, d3-methionine, d4-tyrosine, d3-leucine, d8-phenylalanine, d5-tryptophan, d5-hippuric acid, Cl-phenylalanine, Br-phenylalanine, d5-indole acetate, d9-progesterone, and d4-dioctylpthalate.) were added to each of three aliquots to control the quality of the chromatographic and mass spectrometric analyses. Each of the three aliquots were analyzed via a unique mass spectrometry assay: (1) gas chromatography coupled mass spectrometry (GC-MS) (2) liquid chromatography coupled mass spectrometry in positive ion mode (LC-MS pos), and (3) LC-MS in negative ion mode (LC-MS neg). For GC-MS analysis, analytes were derivatized using bistrimethyl-silyl-trifluoroacetamide and analyzed on a Trace DSQ fast-scanning single-quadruple mass spectrometer (Thermo-Finnigan). For LC-MS analyses one specimen was resuspended in 50 μl of 6.5 mM ammonium bicarbonate, pH 8, for liquid chromatography mass spectrometry (LC/MS) analysis in negative ion mode the other was resuspended in 50 μl of 0.1 % formic acid in 10 % methanol for LC/MS analysis in positive ion mode. Both resuspension buffers contained instrument internal isotopic standards used to monitor performance and serve as retention index markers. Standards for negative ion mode analyses included d7-glucose, d3-methionine, d3-leucine, d8-phenylalanine, d5-tryptophan, Cl-phenylalanine, Br-phenylalanine, d15-octanoic acid, d19-decanoic acid, d27-tetradecanoic acid, and d35-octadecanoic acid. Standards for positive ion mode analyses included d7-glucose, fluorophenylglycine, d3-methionine, d4-tyrosine, d3-leucine, d8-phenylalanine, d5-tryptophan, d5-hippuric acid, Cl-phenylalanine, Br-phenylalanine, d5-indole acetate, d9-progesterone, and d4-dioctylpthalate. Internal standards were chosen based on their broad chemical structures, biological variety and their elution spectrum on each of the arms of the platform. Chromatographic separation was completed using an ACQUITY UPLC (Waters) equipped with a Waters BEH C18 column followed by analysis with an Orbitrap Elite high resolution mass spectrometer (Thermo-Finnigan) (Evans et al 2009). For all analytic methods, metabolites were identified by matching the ion chromatographic retention index, accurate mass, and mass spectral fragmentation signatures with reference library entries created from authentic standard metabolites under the identical analytical procedure as the experimental samples (Dehaven et al 2010).

#### Targeted assays

For quantitative amino acid analysis, plasma samples were acidified, and large molecules were precipitated using equal volumes of Seraprep (Pickering Laboratories) and 200 mM lithium citrate (pH = 2.2). Filtered supernatant was analyzed via cation exchange chromatography using either a Biochrom 30 or Hitachi L-8900 amino acid analyzer. Quantitative acylcarnitine analysis was completed essentially as described previously (Vreken et al 1999). Protein was precipitated using a 50 % acetonitrile solution spiked with a panel of commercially available acylcarnitine stable isotopes (Cambridge isotope, Cat# NSK-B and NSK-B-G). Supernatant was clarified, dried to completion under N_2_, and resuspended in 3 N HCL in n-butanol. Derivatized specimens were analyzed using an Acquity TQ tandem MS (Waters) without chromatographic separation. Quantitative values were calculated through comparison to spiked isotopic reference standards.

#### Data analysis and statistics

**Fig. 1:**
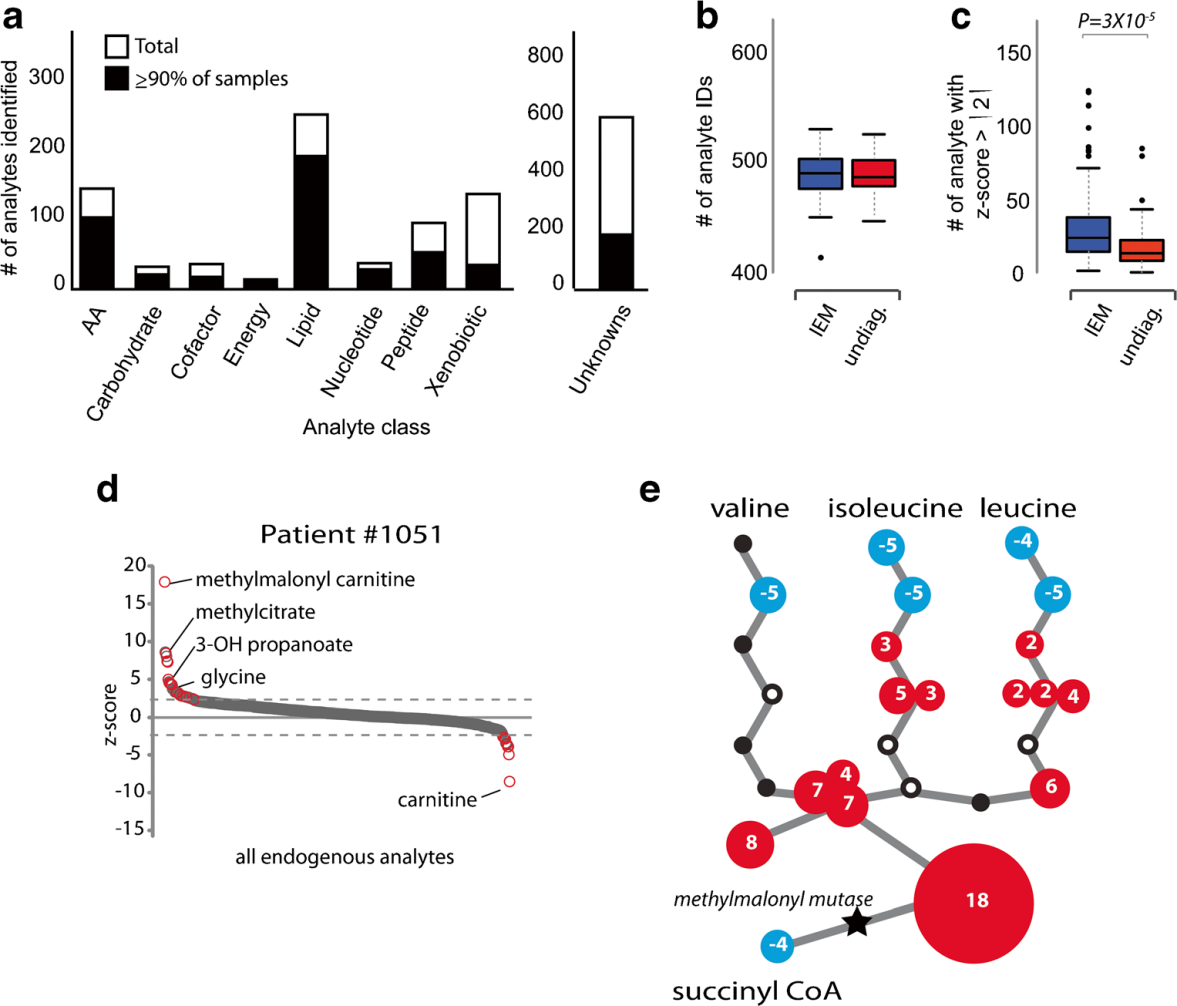
Overview of metabolomic results. **a** The total number of analytes identified for each class of biochemicals is shown. Overlapping black bars indicate the number of analytes identified in 90 % or more of the 190 patient samples tested. The number of unique endogenous analytes were calculated for IEM and undiagnosed patient populations: **b** Boxplots indicate total analyte identifications; **c** total number of analytes with a z-score >2 or < −2. Metabolomic data for patient #1051 with methylmalonyl mutase deficiency (MMA) are shown in **d** and **e. d** All endogenous analyte z-scores were plotted in ranked order; only a subset of significant analytes are named in the plot. **e** Analyte z-score findings for patient #1051 were overlaid onto the branch chain amino acid (BCAA) degradation pathway affected in MMA patients. Each node represents a single metabolic step. Filled *numbered circles* show z-scores for significant analyte findings and the size of the *circle* is proportionate to the z-score with *red dots* representing positive z-scores and *blue dots* negative z-scores. *Black dots* represent analytes with a z-score that was not significant; *open circles* indicate analytes not identified by this analysis. The pathway position of the enzymatic deficiency, methylmalonyl mutase, is indicated by a *black star*. Multiple findings are indicated for some nodes due to the production of conjugated metabolites (e.g., excess propionyl-CoA is converted to propionylcarnitine and propionylglycine). Full BCAA pathway annotation and a more comprehensive example of patient findings related to this pathway can be found in Fig. S5

Raw analyte values correspond to integrated intensity values as calculated using the area under the chromatographic peak. For data analysis, raw values were first median scaled. Next, missing values were imputed using the minimum detected value, and finally the data were log-transformed. Any analytes detected in fewer than 10 % of the samples were excluded from subsequent semiquantitative z-score analysis and were instead classified as “rare analytes” for which a positive identification alone may be clinically relevant. Z-scores were calculated by comparing analyte log transformed median scaled values to the associated mean and standard deviation found in the undiagnosed population (*n* = 70) with one exception; for the analysis of significant z-score findings (Fig. 1), the z-score was calculated using the mean and standard deviation from the entire 190 patient dataset and statistical analysis was completed using a two-tailed heteroscedastic student’s *t*-test. Welch’s two sample *t*- tests were used to compare data obtained from the different disease diagnoses. Multiple comparisons were accounted for with the false discovery (FDR) rate method, and each FDR was estimated by *q*-values. Pearson correlation analysis was completed by comparing the Log_2_ (origscale) raw metabolomic intensity data to the Log_2_ (molar concentrations) found in targeted analysis. To monitor process variability for each batch, the coefficient of variation (CV) was calculated for all spiked standards (listed above) using median scaled values. Overall, spiked extraction standards and instrument internal standards detected a median process variability of ≤5 % in all batches.

## Results

### Overview of analyte findings and reproducibility of metabolomic analyses

In total, we collected 120 specimens from patients with a confirmed IEM, as well as 70 specimens from patients tested for IEMs but with normal results. The sample set consisted of 21 different IEMs, with most disorders represented by >2 unique individuals (Table 1). The majority of diagnosed patients in this study were under clinical management at the time of sampling. For metabolomic analysis, small molecule analytes ranging in size from 50 to 1500 Da were extracted from plasma and subjected to three separate MS analyses run in parallel as described in Methods. Resultant data were annotated using a library containing the chromatographic and spectral signatures of over 2500 metabolites originating from human metabolic processes (hereafter called endogenous analytes). This library also contained many thousands more xenobiotic compounds and unnamed “X” compounds that have unique chromatographic and spectral profiles but are not currently associated with a known analyte.

**Table 1:** Overview of inborn errors of metabolism evaluated in this study

Disorder	Total specimens (unique patients)	Important missing biomarkers	Disease related analytes^¶^
3-Methylcrotonyl-CoA carboxylase (3-MCC) deficiency	4 (4)		3-methylcrotonylglycine, hydroxyisovaleroylcarnitine, beta-hydroxyisovalerate, alpha-hydroxyisovalerate, isovalerylglycine, carnitine(−)
Argininosuccinic acid lyase (AL) deficiency	2 (2)		Citrulline, argininosuccinate(R), creatine(−)
Argininemia	4 (4)		Arginine, N-acetylarginine, homoarginine, 4-guanidinobutanoate, orotate, ornithine(−)
Cobalamin-related disorders	6 (6)	Homocysteine, methylmalonate, cobalamin	2-methylmalonyl carnitine, propionylcarnitine, 2-methylcitrate
Citrullinemia	9 (7)		Citrulline, 3-ureidopropionate, uridine, homocitrulline,
Glutaric aciduria type 1 (GA type1)	5 (5)		Glutaroylcarnitine, glutarate
Guanidinoacetate methyltransferase (GAMT) deficiency	8 (8)	Guanidinoacetate	None
3-OH-3methylglutaryl (HMG) CoA lyase deficiency	2 (2)		3-methylglutaroylcarnitine, hydroxyisovaleroylcarnitine, glutaroylcarnitine, beta-hydroxyisovalerate, isovalerylcarnitine
Holocarboxylase deficiency	1 (1)		3-methylcrotonylglycine, hydroxyisovaleroylcarnitine, beta-hydroxyisovalerate, 3-hydroxypropanoate, propionylcarnitine, 2-methylcitrate, propionylglycine, alanine
Homocystinuria	2 (1)	Homocysteine, S-adenosylmethionine	Methionine, 5-methylthioadenosine, S-adenosylhomocysteine
Isovaleric aciduria	2 (2)		Isovalerylglycine, isovalerylcarnitine, isovalerate, beta-hydroxyisovalerate(−)
Lysinuric protein intolerance (LPI)	2 (2)		Glutamine, lysine(−), ornithine(−), arginine(−)
Maple syrup urine disease (MSUD)	18 (7)		Allo-isoleucine, 4-methyl-2-oxopentanoate, 3-methyl-2-oxovalerate, leucine, 2-hydroxy-3-methylvalerate, isoleucine, alpha-hydroxyisovalerate, 3-hydroxyisobutyrate(−), isovalerylcarnitine (−), 2-methylbutyroylcarnitine(−), hydroxyisovaleroylcarnitine (−)
Medium chain acyl-CoA dehydrogenase deficiency (MCAD)	2 (2)		Hexanoylglycine, N-octanoylglycine, caproate, octanoylcarnitine, hexanoylcarnitine, caprylate, suberate, sebacate, 5-hydroxyhexanoate, (see Table S1 for full list)
Methylmalonic aciduria (MMA)	9 (7)	Methylmalonate	2-methylmalonyl carnitine, propionylcarnitine, 2-methylcitrate, 2-methylbutyroylcarnitine, butyrylcarnitine, tiglyl carnitine, hydroxyisovaleroylcarnitine, glycine, 3-hydroxypropanoate
Ornithine transcarbamoylase deficiency (OTC)	19 (14)		Disease-related findings were mitigated by clinical management at the time of sampling. Instead, treatment-related compounds phenylacetate, phenylacetylglutamine were elevated in the majority of specimens.
Propionic aciduria (PA)	9 (8)		Propionylglycine, 3-hydroxypropanoate, propionylcarnitine, 2-methylcitrate, glycine, hydroxyisovaleroylcarnitine, tiglyl carnitine, 2-methylmalonyl carnitine(−), succinylcarnitine(−)
Phenylketonuria (PKU)	8 (8)		Phenylalanine, phenyllactate, gamma-glutamylphenylalanine, phenylpyruvate, N-acetylphenylalanine
Thymidine phosphorylase deficiency (MNGIE)	2 (2)		Thymidine(R), 2’-deoxyuridine, 5,6-dihydrothymine(−)
Trimethyllysine hydroxylase epsilon deficiency (TMHLE)	4 (4)		N-6-trimethyllysine, deoxycarnitine(−)
Very-long chain acyl-CoA dehydrogenase deficiency (VLCAD)	2 (2)	Tetradecenoylcarnitine, tetradecadienoylcarnitine	Myristoylcarnitine, stearoylcarnitine, palmitoylcarnitine, oleoylcarnitine

^¶^Analytes listed achieved a z-score > │2│in ≥ 50 % of patients with the indicated IEM. (−) indicates analytes that were significantly decreased and (R) indicates rare compounds only detected in patients with a particular IEM

The average total analyte identifications per sample were 886, of which 489 were known endogenous compounds. Many of these compounds (622 of the total and 397 of the endogenous analytes) were positively identified in ≥ 90 % specimens studied. These reproducibly detected analytes represent many classes of biomarkers, including amino acids, carbohydrates, lipids, and nucleotides (Fig. 1a).

To test the intra-assay precision associated with this platform, a single plasma specimen was split into six aliquots and each was independently prepared and analyzed within the same MS batch. Using raw signal intensity values, the coefficient of variation (CV) was calculated for all analytes identified in five or more of the replicates (964 analytes); the median CV was 10.47 % (IQR = 5.55–22.04 %) (Table S1).

For a subset of analytes, we compared metabolomic values to true molar concentrations. This was made possible by the fact that all samples included in this study also had targeted quantitative analysis for selected metabolites in our clinical laboratory. Tests most commonly ordered were plasma amino acids or acylcarnitines (108 and 25 patients, respectively) (Fig S1a). A total of 34 analytes routinely quantified in these panels were also detected by metabolomic profiling. Using a linear regression analysis, we compared metabolomic raw intensity values to molar concentrations and found a strong positive correlation between testing methodologies for nearly all compounds across a wide range of detection (median *r* = 0.9, IQR *r* = 0.84–0.95; Fig S1b–d), and the breadth of analyte values reported for each testing methodology was roughly equivalent in most cases as estimated by the slope (m) of the regression model (median slope =0.9, IQR slope =0.67–1.19). One major exception, carnitine, had no correlation between platforms, partially as a result of low outlier values in a subset of metabolomic analyses (Fig. S1d).

### Single patient metabolomic analysis can be applied to the detection of a wide range of IEMs

We next inspected the metabolomic data for key biochemical signatures in known IEM patients. All 21 disorders included in this study can be diagnosed via the measurement of small molecule perturbations in plasma, although diet and clinical management can sometimes suppress biochemical findings (Table 1). To help focus our analysis to clinically relevant perturbations, we restricted our initial inspection of patient metabolomic profiles to endogenous analytes that met a z-score cutoff of >2 or < −2. Although total analyte identifications were not significantly different between patients with or without a diagnosed IEM (Fig. 1b), these two groups did have significant differences in the number of analytes achieving the z-score cutoff (median number for IEM patients = 30.9 and undiagnosed patients = 18.7; *p*-value = 2.9 × 10^−5^; Fig. 1c).

**Fig. 2:**
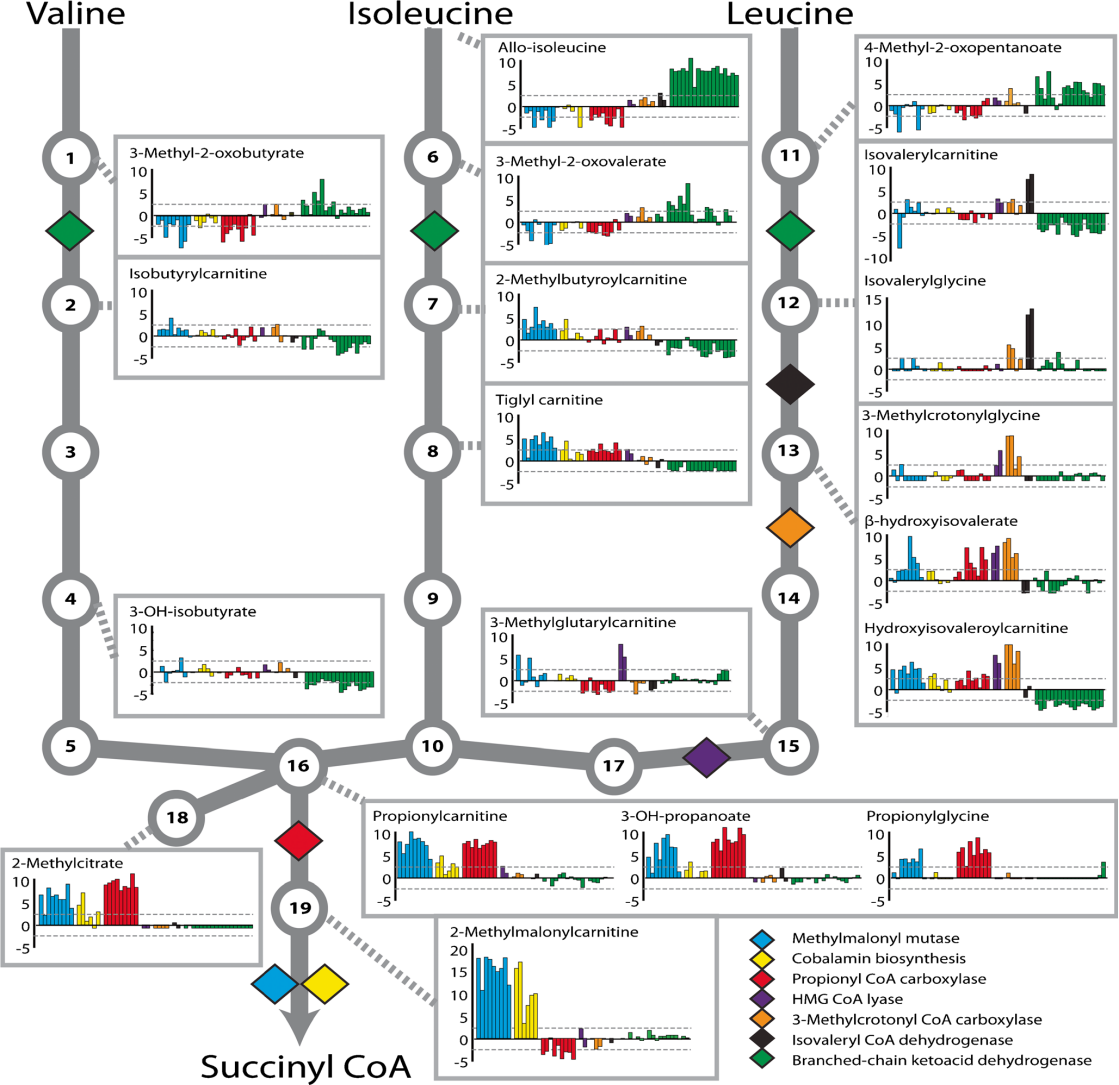
Comprehensive pathway analysis provides useful diagnostic information for branched chain amino acid metabolism. *Bar charts* are shown for a representative subset of metabolites in the branched chain amino acid pathway. Values are given as z-scores (y-axis) and *dashed gray lines* indicate clinically relevant z-score cutoffs (>2 or < −2). Each *bar* represents a unique patient specimen, and each *color* represents the patient’s diagnosis (*blue* = methylmalonic aciduria, *yellow* = cobalamin biosynthesis disorders, *red* = propionyl CoA carboxylase deficiency, *purple* = HMG CoA lyase deficiency, *orange* = 3-methylcrotonyl CoA carboxylase deficiency, *black* = isovaleryl CoA dehydrogenase deficiency, and *green* = branched-chain ketoacid dehydrogenase deficiency). For each disorder, a *colored diamond* pinpoints the pathway position of the deficient enzyme. *Numbered circles* indicate intermediate analytes, **1** 3-methyl-2-oxobutyrate, **2** isobutryl-CoA, **3** methylacrylyl-CoA, **4** 3-OH-isobutryl-CoA, **5** methylmalonic semialdehyde, **6** 3-methyl-2-oxovalerate, **7** 2-methylbutyryl-CoA, **8** tiglyl-CoA, **9** 2-methyl-3-OH-butyryl-CoA, **10** 2-methylacetoacetyl-CoA, **11** 4-methyl-2-oxopentanoate, **12** isovaleryl-CoA, **13** 3-methylcrotonyl CoA, **14** 3-methylglutaconyl CoA, **15** 3-OH-3-methylglutaryl-CoA, **16** propionyl-CoA, **17** acetyl-CoA, **18** methylcitrate, and **19** methylmalonyl-CoA. As with standard clinical testing, many CoA conjugated pathway intermediates listed here are assayed through the measurement of carnitine or glycine conjugated forms

Using this scoring metric, 20 of the 21 disorders had multiple analyte perturbations within the pathway affected by the respective enzyme deficiency (Table 1). We were unable to screen for gaunidinoacetate methyltransferase (GAMT; OMIM 612736) deficiency due to a failure to detect guanidinoacetate, the diagnostic biomarker found elevated in the plasma of patients with this disorder. The number of significantly altered analytes related to the diagnosis ranged from two metabolites in patients with trimethyllysine hydroxylase epsilon (TMLHE; OMIM 300872) deficiency, i.e., decreased deoxycarnitine (γ-butyrobetaine) and increased N6-trimethyllysine (Fig. S2), to as many as 20 significant analyte perturbations that could be mapped directly up/downstream of the enzymatic defect in patients with methylmalonic acidemia (MMA; OMIM 25100; Figs. 1d and e; 2).

**Fig. 3:**
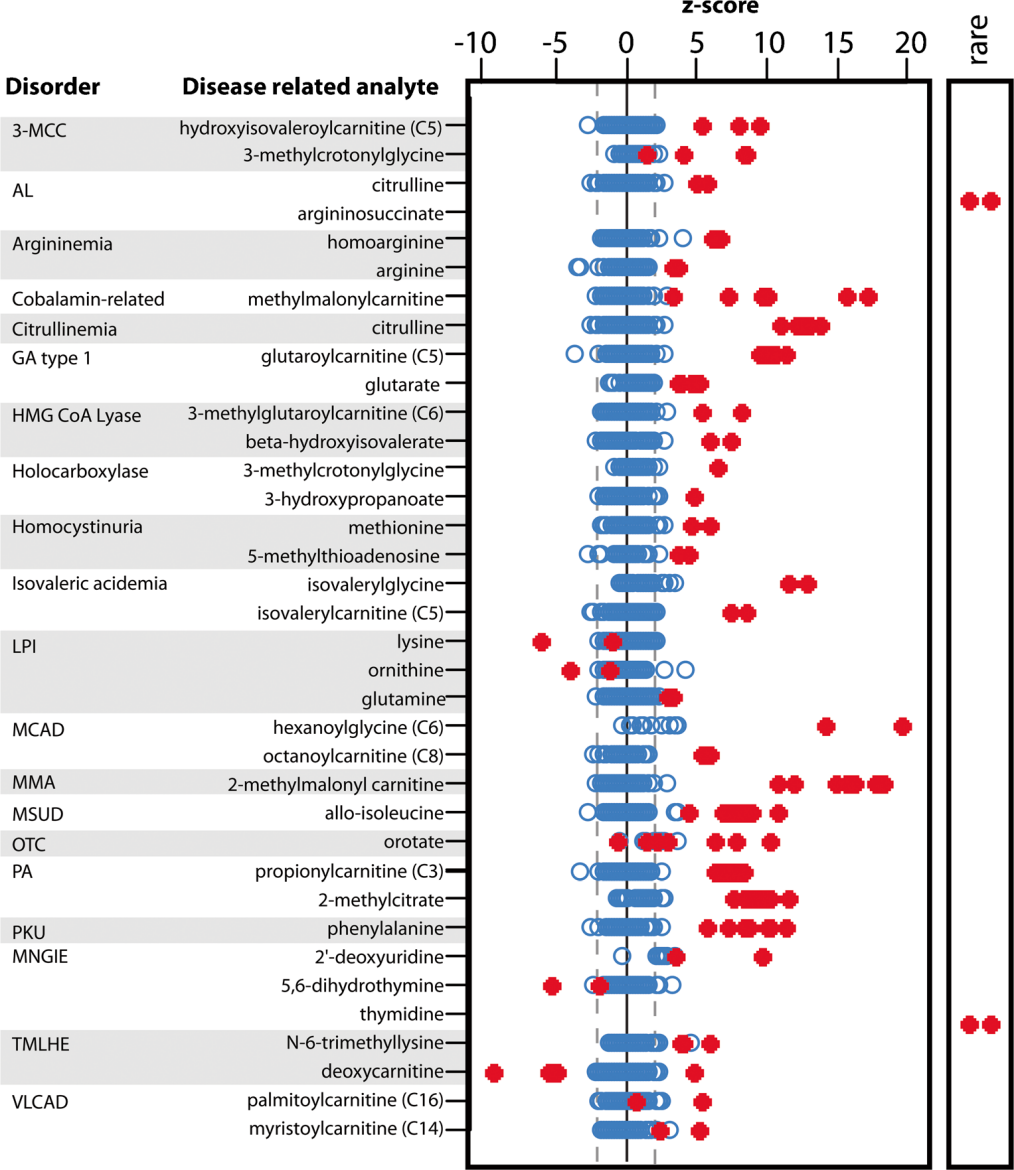
Representative examples of clinically relevant findings for each *inborn error* of metabolism evaluated in this study. Each *dot* represents a unique patient (normal = hollow *blue circles* and IEM patient = filled *red circles*). “Rare analyte” refers to analytes only detected in the indicated patients. See Supplemental Tables 1 and 2 for a full listing of clinically relevant findings for each disorder. See Table 1 for the full name for each disorder

As a means of highlighting key findings and exploring the false positive rate associated with this test, we plotted patient values for one or two of the classic biomarkers for each disorder and compared them to the findings in the undiagnosed patient population (*n* = 70) (Fig. 3). Specific examples include significant elevations of (1) hexanoylglycine in 2 of 2 patients with medium chain acyl-CoA dehydrogenase (MCAD; OMIM 201450) deficiency, (2) alloisoleucine in 7 of 7 patients with maple syrup urine disease (MSUD; OMIM 248600), (3) methylmalonylcarnitine in 7 of 7 patients with MMA, and (4) glutarate in 4 of 4 patients with glutaric aciduria type 1 (GA1; OMIM 231670). In general, there were few analyte outliers in the undiagnosed population (z-score > 2), and most analytes showed clear segregation between affected and unaffected patient populations. For two different IEMs, pathognomonic findings were provided through the identifications of rare analytes. Thymidine was only detected in the two patients with thymidine phosphorylase deficiency, and argininosuccinic acid was found exclusively in the two patients with argininosuccinic acid lyase deficiency (Fig. 3).

Presumptive treatment-related findings were also noted for many patients. Examples include but were not limited to (i) phenylbutyrate and/or sodium benzoate associated analyte elevations in patients with urea cycle disorders, (ii) trimethylamine N-oxide elevations in patients receiving supplemental carnitine (likely due to gut microbial degradation of carnitine) (Koeth et al 2013), (iii) medium chain fatty acid elevations in patients with long chain-related fatty acid oxidation disorders likely supplemented with medium-chain triglyceride (MCT) oil, (iv) creatine elevations in patients presumably receiving treatment with creatine for GAMT deficiency, and (v) numerous pharmaceutical-related metabolites (Fig. S3 and Table S1).

### Additional applications of metabolomic data to uncover phenotypic heterogeneity and identify novel biomarkers

**Fig. 4:**
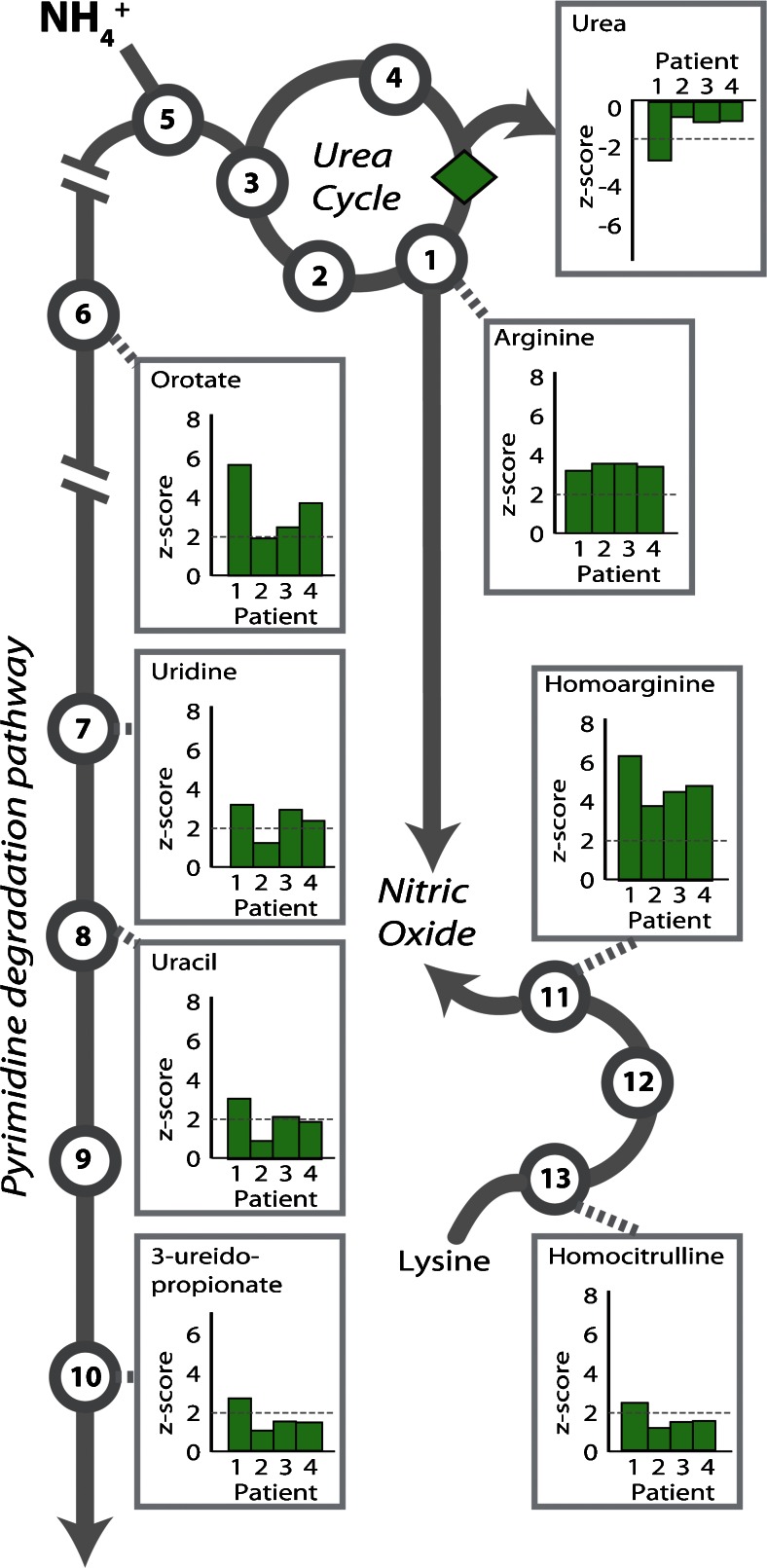
Arginase deficiency reveals significant perturbations in distal pathways. The *green diamond* indicates the location of arginase, the deficient enzyme in these patients. Values are given as z-scores (y-axis) and *dashed gray lines* indicate clinically relevant z-score cutoffs (>2 or < −2). Patients 1–4 correspond to patient #1006–1009, respectively, in Supplemental Table 1. Pathway intermediates are indicated by *numbered circles*, **1** arginine, **2** argininosuccinate, **3** citrulline, **4** ornithine, **5** carbamoylphosphate, **6** orotate, **7** uridine, **8** uracil, **9** dihydrouracil, **10** 3-ureidopropionate, **11** homoarginine, **12** homoargininosuccinate, and **13** homocitrulline

We were next interested in whether the additional analyte information provided by metabolomic analysis might help to better predict phenotypic heterogeneity between patients with the same disorder. Within our sample set were four argininemia (OMIM 207800) patients, all were female and between the ages of 16 to 24 years of age. Argininemia is caused by autosomal recessive mutations affecting the urea cycle enzyme arginase 1, and this disorder is diagnosed via the measurement of plasma arginine levels (Prasad et al 1997). Quantitative plasma amino acid analysis was completed as part of routine clinical management for these patients and all had comparable elevations of arginine in the absence of other significant findings (Table S2). Metabolomic analysis of the same specimen showed similar elevations of arginine in all four patients, but when compared to patients #2–4, patient #1 had much more striking perturbations of more distal compounds such as urea, orotate, and homoarginine (Fig. 4). The more severe biochemical phenotype in this patient was consistent with the clinical findings; in the 3 months prior to sampling patient #1 had been admitted to the hospital on three separate occasions for hyperammonemic events and was receiving sodium benzoate treatment at the time of sampling whereas patients #2–4 had more modest perturbations in distal compounds, had not been admitted to the hospital in the 3 months prior to sampling, and were not on sodium benzoate treatment.

To identify novel biomarkers, data from multiple different patients were pooled and then compared to the undiagnosed population using both a Welch’s two sample *t* test, as well as a false discovery rate calculation to account for multiple sampling. As an example of novel biomarker findings from this approach, phenylketonuria (PKU; OMIM 261600) patients (*n* = 8) had 15 different disease-related metabolomic findings that met our z-score cutoff, including the classic analyte elevations associated with this disorder (e.g., phenylalanine and phenylpyruvate), as well as novel biomarkers not previously described in the plasma of PKU patients (e.g., phenylalanylphenylalanine and phenylalanylleucine) (see Table S3 for similar analyses for multiple other disorders).

## Discussion

In the preceding work we describe an untargeted metabolomic approach capable of screening for a wide variety of IEMs. Overall, we successfully screened for 20 of 21 IEMs on the basis of both the magnitude of key analyte perturbations, as well as the overall pattern of findings within known metabolic pathways. Given the breadth of analytes identified, it is likely many more IEMs could be detected with this platform. Results reported herein show consistent detection of key biomarkers across many specimens with clear segregation of disease related analyte levels between unaffected vs. affected individuals in nearly all cases tested. The data analysis approach we used can be applied to individual specimens without prior establishment of disease-specific reference populations. Therefore, in theory this platform could be used as an initial screen for a wide range of IEMs, as long as there was prior knowledge of the metabolic pathway and the disorder caused significant analyte perturbations.

The key to our data analysis approach was the use of z-scores. Using this metric, we captured the population and technical variation for each analyte and were able to employ biochemical pathway information to uncover diagnostic patterns of analyte perturbations. The cutoff we set (z-score ≥ |2|) does not guarantee statistical significance and instead was chosen to preserve important disease related findings while allowing for significant reduction of data complexity (for the average undiagnosed individual, ~19 analytes met this cutoff (Fig. 1c)). Using a Bonferroni correction to control the family-wise error rate (where *α* = 0.05 and *n* = 500) sets the significance threshold at *p* < 0.0001; this is equivalent to a z-score ≥ |4|. Therefore a z-score ≥ |4| by itself should be strong evidence of a clinically relevant perturbation; nearly all affected patients in our study had one or more disease related analyte that met this more stringent cutoff criterion (Table S1). Additional information can be gained from looking at patterns of more moderate perturbations within a pathway. For instance, moderate decreases, as opposed to striking increases, of methylmalonylcarnitine could be used to distinguish propionic acidemia patients from methylmalonic acidemia patients. As we study additional undiagnosed/untreated patients, more sophisticated ratios or pattern recognition approaches may be developed to automate analysis or allow for more sensitive detection of disorders. Future clinical applications of this technology should pair expert analysis with thorough clinical notes, and diagnostic metabolomic findings should be confirmed with second-tier testing.

Limitations of this platform were also uncovered. A number of clinically relevant plasma compounds were not identified in this study, most notably homocysteine, methylmalonic acid, tetradecenoylcarnitine (C14:1), and guanidinoacetate (GAA). The latter missed identification led to the failure to detect GAMT deficiency; however, the inability to detect homocysteine, tetradecenoylcarnitine, and methylmalonic acid was mitigated by the identification of multiple other disease related analyte perturbations in the case of homocystinuria or methylmalonic acidemia. Given the sample preparation methods, it is understandable that we did not detect homocysteine because analysis requires a reducing reagent to release conjugated homocysteine (Frick et al 2003). Overall, metabolomic analysis provided excellent coverage of the amino acids and acylcarnitines covered by targeted clinical tests (Tables S4 and S5); however, long chain hydroxylated acylcarnitines were missed and therefore this platform would likely not be able to diagnose trifunctional protein deficiency (OMIM 609015). For many OTC deficiency patients, only treatment-associated findings were uncovered, similar to the reports for clinical amino acid testing of the same specimens. Thus, as with targeted assays, metabolomic results have to be interpreted with the caveat that a patient’s diet and physiological condition may mask the underlying genetic disorder. It is worth reiterating that almost all specimens in our study were collected while the patient was on clinical management designed to alleviate their biochemical phenotype. For instance, citrulline and/or arginine levels would likely be significantly reduced in OTC patients if they were not receiving supplementation of these amino acids. In many cases, but especially in the OTC cohort, our dataset likely does not fully demonstrate the capabilities of this platform in a clinical diagnostic setting focused on undiagnosed patients.

While the results of this initial proof of concept study are encouraging, there are additional technical considerations not addressed in this manuscript. Further studies are needed to test the reproducibility of this platform across multiple independent analyses completed across a wide time frame. Additional quantitative analyses using spiked isotopic standards would also be beneficial to explore the reference ranges of analytes not tested in our correlative analysis of amino acids and acylcarnitines; this analysis would be especially important for the subset of clinically relevant analytes found to have poor intra-assay precision.

For the 70 undiagnosed patients in this study, metabolomic analysis failed to uncover a new biochemical diagnosis that could explain a patient’s phenotype. However, metabolomic findings for two patients, #1155 and #1176, were highly suspicious for biochemical disorders that were likely unrelated to clinical findings, TMLHE deficiency and sarcosinemia (OMIM 268900), respectively (Fig. S4). In two other cases, the metabolomic results uncovered ornithine transcarbamylase deficiency (OMIM 311250) female heterozygotes that were misassigned to the undiagnosed population (patients #1136 and #1166), based upon normal clinical plasma amino acid results. Suspicion of an OTC diagnosis was raised by elevations (z-score >2) of the pyrimidine pathway intermediates uracil, uridine, orotate, and 3-ureidopropionate, as well as glutamine, in patient #1136 and phenylacetylglutamine and phenylacetate elevations in patient #1166 caused by phenylbutyrate treatment. Diagnosis in these cases was confirmed through examination of the clinical record and consultation with the attending physician.

The primary advantage of untargeted metabolomic analysis over traditional targeted assays relates to the scope of detections possible. A single metabolomic analysis can provide information that currently would require multiple different clinical tests, including targeted panel assays for amino acids, acylcarnitines, organic acids, purines, pyrimidines, acylglycines, bile acids, and carnitine biosynthesis intermediates. In addition, we estimate that >400 endogenous analytes identified in this study could not be detected in our clinical biochemical genetics laboratory even when using the full repertoire of tests available to us, including urine analyses. Therefore, plasma metabolomic analysis may be an attractive alternative when the patient’s phenotype is undifferentiated (e.g., seizures, intellectual disability, or recurrent vomiting) and the clinician is considering ordering a combination of biochemical genetics tests, especially those for plasma amino acids, plasma acylcarnitines, and urine organic acids. One option would be to start with metabolomic testing and selected other tests such as homocysteine, followed by phenotype specific testing for disorders that might be missed by metabolomic analysis. Through this approach metabolomic analysis may expedite diagnosis while allowing for the possibility to identify novel biomarkers, IEMs, and/or sources of phenotypic heterogeneity within a particular disease.

## Electronic supplementary material

Below is the link to the electronic supplementary material.

ESM 1 (XLS 4525 kb)
